# Characterisation of a subpopulation of CD133^+^ cancer stem cells from Chinese patients with oral squamous cell carcinoma

**DOI:** 10.1038/s41598-020-64947-9

**Published:** 2020-06-01

**Authors:** Zhen Ma, Chong Zhang, Xiaotong Liu, Fang Fang, Shiqi Liu, Xianxiang Liao, Shicheng Tao, Huaming Mai

**Affiliations:** 1 Department of Oral and Maxillofacial Surgery, College and Hospital of Stomatology, Guangxi Medical University, Nanning, Guangxi 530021 China; 2 Guangxi Key Laboratory of Oral and Maxillofacial Rehabilitation and Reconstruction, Nanning, Guangxi 530021 China; 3 Guangxi Key Laboratory of Oral and Maxillofacial Surgery Disease Treatment, Nanning, Guangxi 530021 China; 4 Guangxi Clinical Research Center for Craniofacial Deformity, Nanning, Guangxi 530021 China

**Keywords:** Cancer stem cells, Oral cancer

## Abstract

Cancer stem cells (CSCs) play a critical role in cancer development and growth. The aim of this study was to identify and isolate CSCs from populations of primary oral squamous cell carcinoma (OSCC) cells, which were obtained from OSCC specimens and identified by cell morphology and immunohistochemical staining for keratin. CD133^+^ cells detected by flow cytometry comprised 0.41 ± 0.06% of primary OSCC cells and were isolated from primary OSCC cell populations using magnetic-activated cell sorting, revealing that 93.39% of high-purity CD133^+^ cells were in the G0/G1 phase of the cell cycle. Additionally, the growth rate of CD133^+^ cells was higher than that of CD133^−^ cells, and *in vivo* tumourigenesis experiments showed that the tumourigenic ability of CD133^+^ cells was markedly stronger than that of CD133^−^ cells. Moreover, CD133^+^ cells showed increased chemotherapeutic resistance to cisplatin and higher self-renewal ability according to sphere-formation assay, as well as higher mRNA levels of stemness-associated genes, including *NANOG*, *SOX2*, *ALDH1A1*, and *OCT4*. These results indicated that OSCC cells, which share certain characteristics of CSCs, harbour CD133^+^ cells potentially responsible for OSCC aggressiveness, suggesting CD133 as a potential prognostic marker and therapeutic target.

## Introduction

Head and neck cancer (HNC) is the sixth most common malignancy worldwide, with oral squamous cell carcinoma (OSCC) the most common type of HNC and accounting for >90% of all oral cancers^1,2^. Smoking, drinking, and chewing areca nuts are considered risk factors for OSCC, which can affect any part of the mouth, including the lips, tongue, and throat^3^. Furthermore, when patients develop OSCC, they experience maxillofacial deformity and psychological trauma in addition to common symptoms of cancer. Although there have been advances in surgical techniques and chemotherapeutic strategies for OSCC, the patient survival rate remains low^4^. Cisplatin is the first-line chemotherapeutic drug currently used to treat patients with locally advanced, resectable OSCC; however, cisplatin resistance poses a major challenge for its clinical application^5^ and is considered the critical cause of tumour recurrence in OSCC patients^6^. Therefore, a better understanding of the mechanisms underlying OSCC recurrence is required to develop novel therapeutic strategies^7^.

Studies have increasingly focused on the roles of cancer stem cells (CSCs) in cancer invasion and recurrence^8^. CSCs represent highly heterogeneous subpopulations with functional heterogeneity in their respective tumours and are characterised by infinite proliferation, self-renewal, chemotherapeutic resistance, and multidirectionality^9^. The role of CSCs was previously demonstrated in the development and therapeutic resistance of liver cancer^10^. Similar to their roles in other malignancies, CSCs also play a pivotal role in OSCC development and progression^11,12^. The study of CSCs, also referred to as tumour-initiating cells^13^, has recently become among the most popular research directions in cancer biology. Cell plasticity and the ability of CSCs to adapt in the G0/G1 phase of the cell cycle have emerged as important drivers of chemoresistance^14^. Therefore, targeted CSC treatment might represent an effective way to accelerate tumour cell death and reduce chemoresistance.

CD133 (prominin-1) is found in epithelial cells and associated with many solid tumours, including those related to prostate carcinoma, thyroid carcinoma, hepatoma, renal tumours, and oral cancer^15–18^. A recent study of colon cancer reported that CD133^+^ cells show strong clonality, tumour-colony formation ability, and xenograft tumourigenic ability^19^. Additionally, CD133 is negatively correlated with the survival prognosis of OSCC patients^20^. Moreover, CD133^+^ cells show a greater ability to self-renew and differentiate than CD133^−^ cells and undifferentiated tumour cells, suggesting that CD133 might be a surface marker of CSCs. A previous study of OSCC reported that human OSCC cell lines, such as CAL27 and SCC9, express CD133^21^; however, these cell lines were mostly derived from patients of European descent, with only a few reports exploring CD133 in a Chinese OSCC cell line (Tca8113) and suggesting that a larger cohort needs to be studied^22,23^. The objective of this study was to isolate and characterise CSCs from primary OSCC cells using CD133 as a CSC marker. *In vitro* and *in vivo* results showed that stemness, adhesion, motility, and proliferation were significantly increased in the CD133^+^ subpopulation, with these cells fully capable of self-renewal and serial propagation of tumours in BALB/c mice. These results demonstrate that CD133 can discriminate a specific OSCC cell subset that sustains cancer stemness and promotes tumour formation and chemoresistance.

## Results

### **Subpopulations of CD133**^**+**^**cells in OSCC**

**Figure 1:**
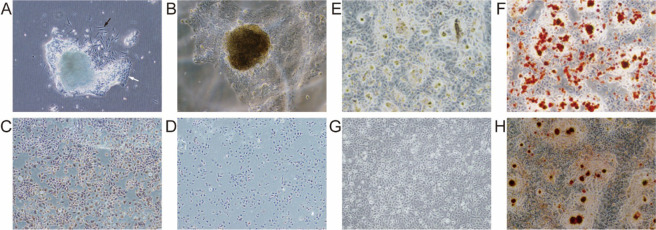
OSCC cell features. (**A**) OSCC cells (black arrow) and fibroblasts (white arrow) direct outgrowth from the OSCC specimens. (**B**) OSCCs after a 15-day culture. (**C**) Brown staining was positive in the cytoplasm of OSCC cells detected by immunohistochemical staining for keratin. (**D**) Minimal brown staining was observed in the cytoplasm of OSCC cells in the blank control group. (**E,F**) CD133^+^ OSCC cells formed in adipose tissue observed by microscopy following Oil Red O staining. (**G,H**) Calcified nodules formed by CD133^+^ OSCC cells stained with Alizarin Red.

A large number of primary OSCC cells were obtained and successfully passaged. After 3 days of culture, OSCC cells and fibroblasts were observed around the tissue (Fig. 1A), and after 15 days, cancer cells grew to 80% confluence (Fig. 1B). We then used differential digestion with trypsin to obtain purified OSCC cells in passage 4. Immunohistochemical staining for keratin showed positive brown staining in the cytoplasm of OSCC cells, whereas minimal brown staining was observed in the cytoplasm of the OSCC blank control group, in which phosphate-buffered saline (PBS) was used instead of a rabbit anti-CK3 antibody (Fig. 1C,D). The results of adipogenic and osteogenic differentiation assays indicated that CD133^+^ CSCs showed an ability to form adipose and bone tissues, with bright and transparent adipose tissue observed by microscopy following Oil Red O staining (Fig. 1E,F). Additionally, calcified nodules formed by CD133^+^ CSCs were stained with Alizarin Red (Fig. 1G,H).

### **Magnetic-activated cell sorting (MACS) and characterisation of CD133**^**+**^**OSCC cells**

**Figure 2:**
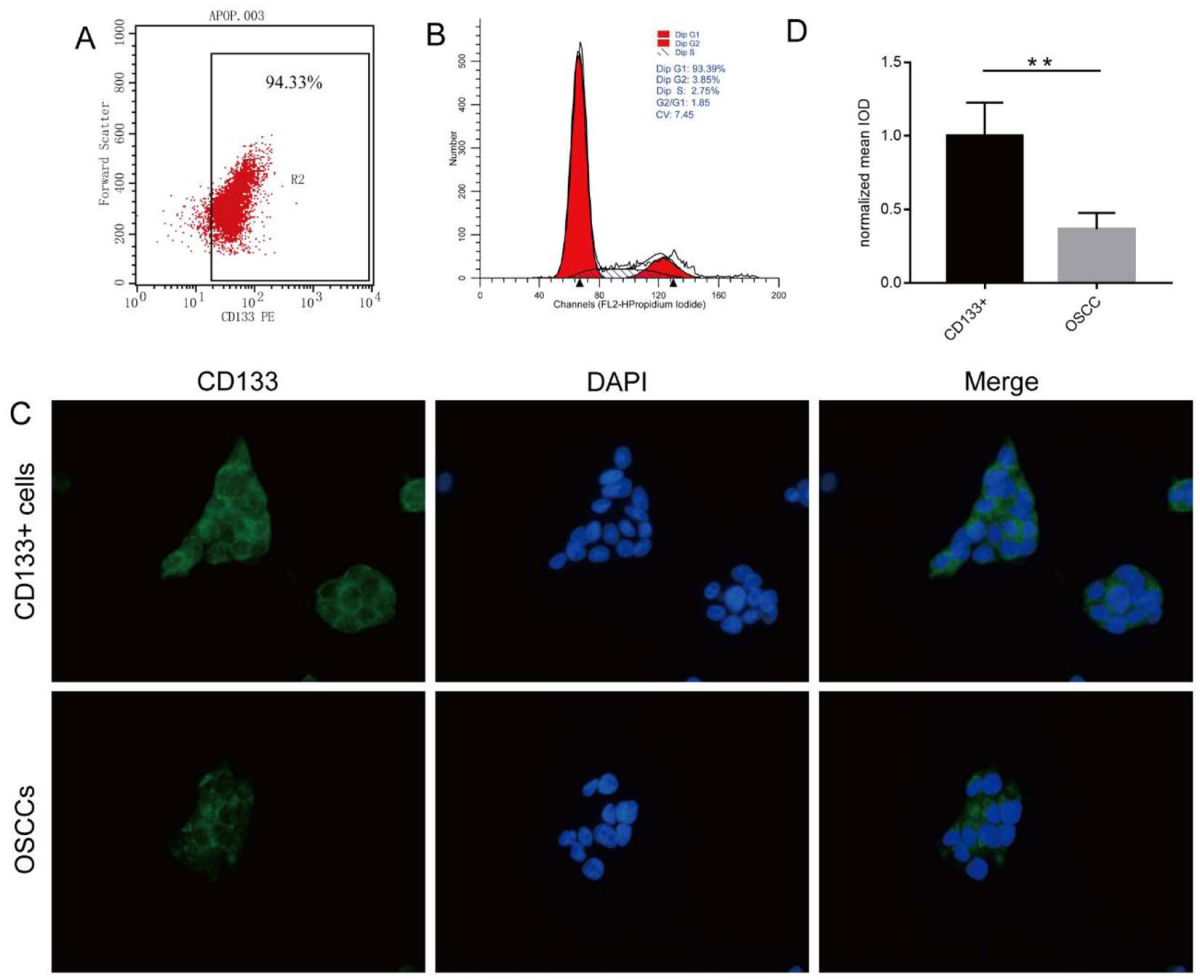
Characterization of CD133^+^ OSCC cells. (**A**) CD133^+^ cells purified up to 94.33% using MACS. Expression of CD133 in 0.41%±0.06 of primary OSCC cells and detected by flow cytometry. (**B**) Cell cycle analysis of CD133^+^ cells revealing that 93.39% of CD133^+^ OSCCs cells were in the G0/G1 phase. (**C**) Immunofluorescence detection of CD133 expression on OSCC cells and primary OSCC cells. All images were obtained at 400× magnification. (**D**) The normalized mean IOD of CD133^+^ OSCC cells was higher than that of primary OSCC cells. Data were analysed by 2-tailed *t* test. **P < 0.01.

**Figure 3:**
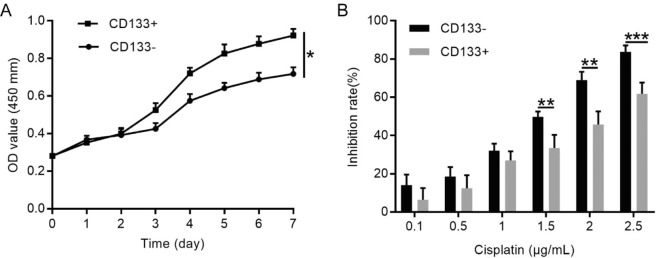
Self-renewal ability and chemotherapy resistance of CD133^+^ and CD133^−^ OSCC cells. (**A**) The growth curve of CD133^+^ cells was significantly higher than that of CD133^−^ cells. (**B**) CD133^+^ cells displayed greater chemotherapy resistance to cisplatin as compared with CD133^−^ cells. Data were analysed by 2-tailed *t* test. *P < 0.05.

Flow cytometry showed that 0.41 ± 0.06% of primary OSCC cells expressed CD133, and that 33.76 ± 25.34% primary OSCC cells expressed CD44, respectively (see Supplementary Table S1 online). MACS allowed purification of CD133^+^ cells to up to 94.33% (Fig. 2A). Cell cycle analysis showed that 93.39% of CD133^+^ CSCs were in the G0/G1 phase, which was consistent with the characteristics of stem cells (Fig. 2B). To investigate whether CD133 expression is also altered *in vivo*, we performed immunofluorescence analysis of CD133^+^ and primary OSCC cells (Fig. 2C), finding that the normalised mean integral optical density of CD133^+^ cells was higher than that of primary OSCC cells (*P* < 0.01) (Fig. 2D). Additionally, the expression of CD133 on OSCC cells led us to hypothesise that CD133 contributed to the stemness properties of CD133^+^ OSCC cells. MACS-sorted CD133^+^ and CD133^−^ cells were then subjected to further phenotypic characterisation, including a cell proliferation assay. The results showed that the growth rate of CD133^+^ cells was significantly higher than that of CD133^−^ cells (Fig. 3A), with the doubling times of CD133^+^ and CD133^−^ cells at 98.30 ± 3.89 h and 124.10 ± 6.93 h, respectively (*P* < 0.001).

### **Resistance of CD133**^**+**^**OSCC cells to chemotherapy. Because cisplatin treatment is a major clinical criterion for OSCC, we evaluated the chemosensitivity of CD133**^**+**^**and CD133**^**−**^**cells**

To identify the optimal concentrations for treatment, the IC_50_ values of cisplatin were determined at 1.5 µg/mL for CD133^−^ cells and 2.5 µg/mL for CD133^+^ cells. Additionally, CD133^+^ cells showed a significantly greater resistance to cisplatin than did autologous CD133^−^ cells (*P* < 0.05) (Fig. 3B). These findings suggested that CD133^+^ cells might be critical for modulating OSCC chemosensitivity.

### **Self-renewal and sphere-forming abilities of CD133**^**+**^**and CD133**^**−**^**OSCC cells. Formation of ultra-low-adhesion spherical clusters of cells or spheres in serum-free culture is a CSC characteristic**

**Figure 4:**
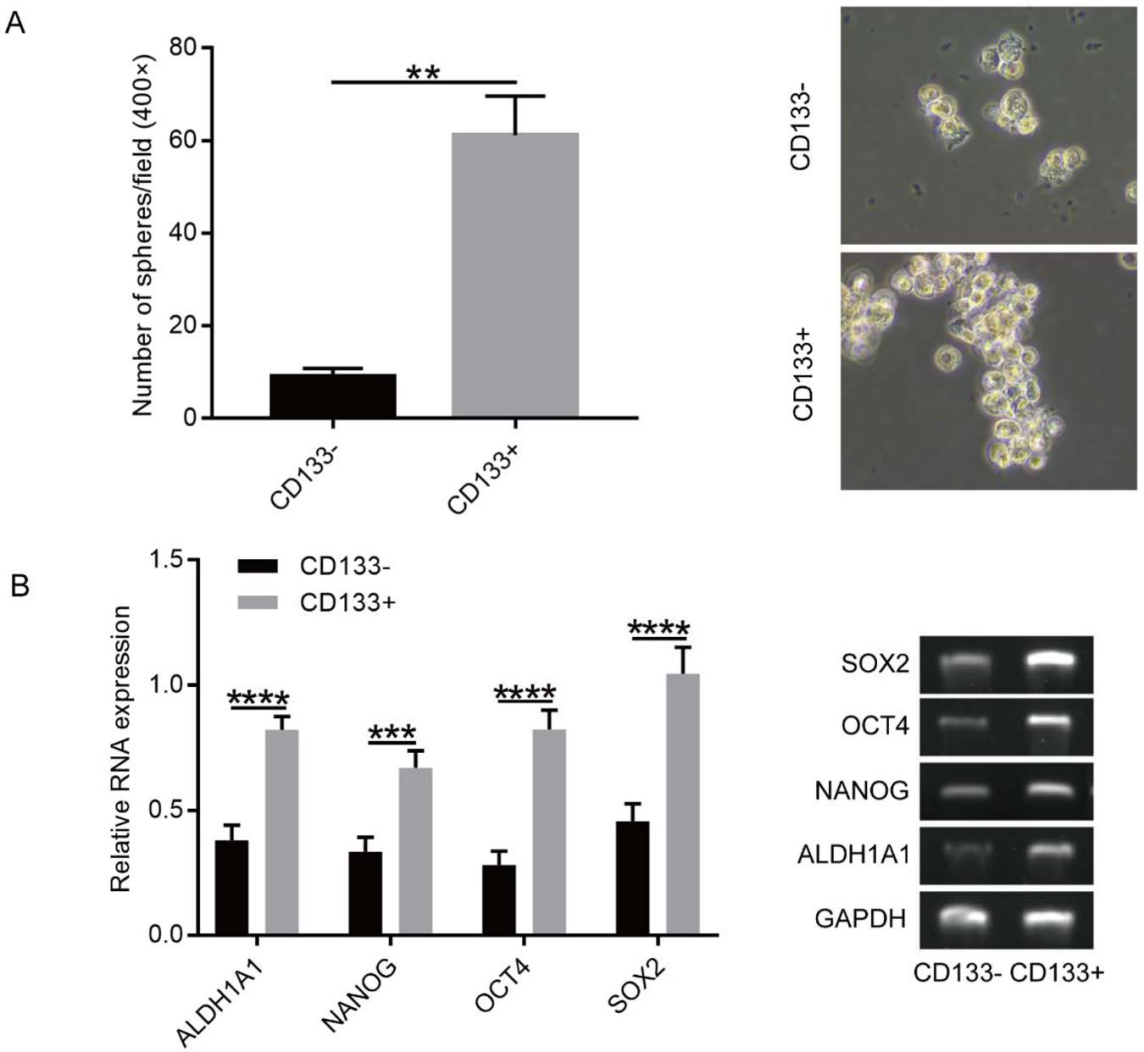
Sphere-formation assay and RT-PCR analysis. (**A**) More spheroids were observed in cultures of CD133^+^ cells as compared with cultures of CD133^−^ cells, Magnification, 400×. Data were analysed by 2-tailed *t* test. **P < 0.01. (**B**) CD133^+^ cell populations showed higher mRNA levels of stemness-associated genes. Data were analysed by 2-tailed *t* test. ***P < 0.001.

We observed significantly more clones, as well as larger spheroids, in cultures of CD133^+^ cells than those of CD133^−^ cells (*P* < 0.01) (Fig. 4A). This suggested that CD133^+^ cells isolated by MACS from primary OSCC cells had a tendency to form spheres more efficiently than the CD133^−^ cells.

### **Gene expression of CSC markers**. **After demonstration of the ability of tumour-derived cells to form spheroids possessing higher tumourigenic potential, we evaluated levels of stemness-associated genes in CD133**^**+**^**and CD133**^**−**^**cells**

Reverse transcription polymerase chain reaction (RT-PCR) revealed that CD133^+^ cell populations showed higher mRNA levels of stemness-associated genes, including *NANOG*, *SOX2*, *ALDH1A1*, and *OCT4*, than CD133^−^ cells (*P* < 0.05) (Fig. 4B), suggesting that CD133^+^ cells from OSCC tumours showed more aggressive tumour stem cell behaviour.

### **Tumourigenic abilities of CD133**^**+**^**and CD133**^**−**^**OSCC cells in mice**

**Figure 5:**
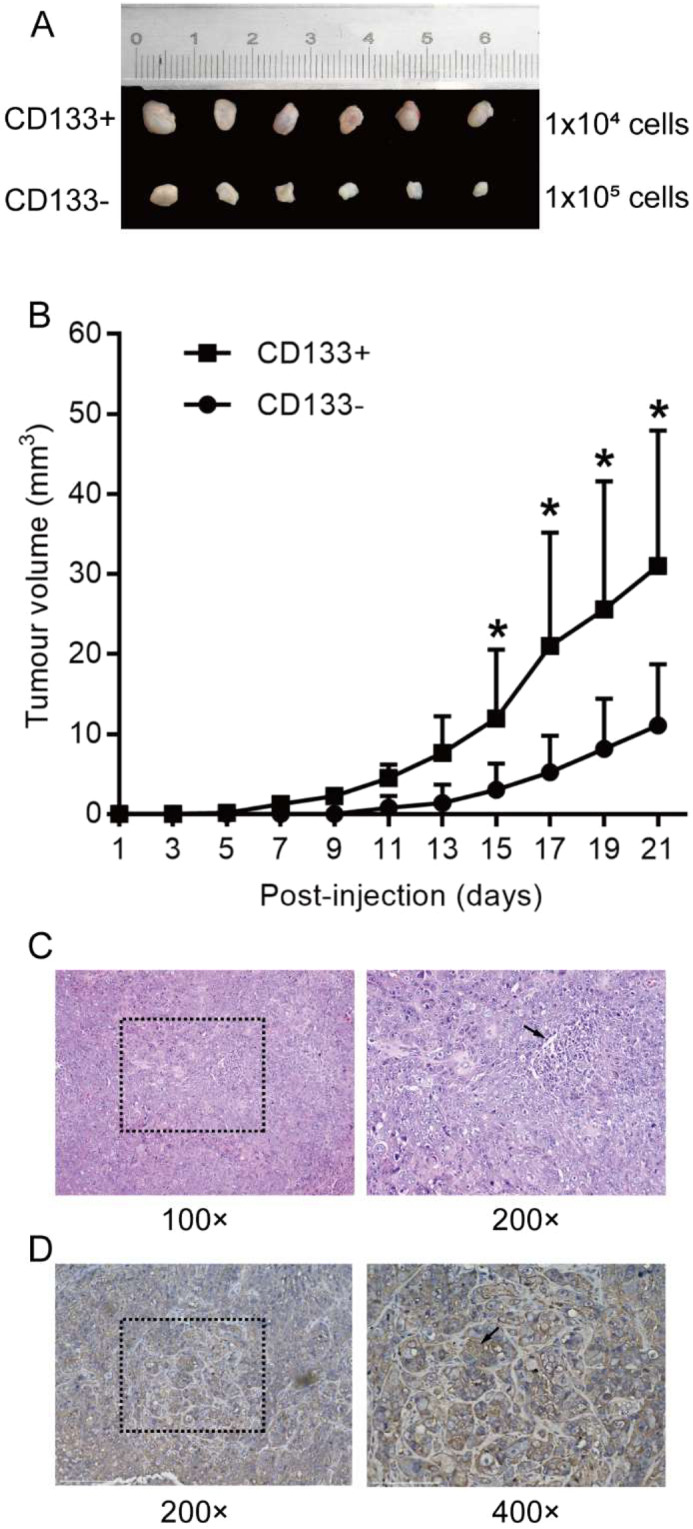
*In vivo* tumourigenic assay. (**A,B**) CD133^+^ OSCC cells showed more aggressive tumour stem cell behaviour. Data analysed by 2-tailed *t* test. *P < 0.01. (**C**) The transplanted tumour was identified as well-differentiated SCC according to HE staining. Cancer pearls observed in the specimen (**C**, black arrow). (**D**) Cytokeratin detected in the CD133^+^-specific transplanted tumour according to immunohistochemical staining (black arrow).

To determine whether CD133^+^ cells were more tumourigenic than CD133^−^ cells, purified cells were subcutaneously injected into BALB/c mice. We observed a markedly higher tumour incidence at 3 weeks post-injection of CD133^+^ cells than that following CD133^−^ cell injection (*n* = 6; *P* < 0.05) (Fig. 5A,B). This suggested that CD133^+^ cells showed more aggressive CSC-like behaviour. Additionally, we confirmed that the transplanted tumours were highly differentiated SCCs according to haematoxylin–eosin (HE) staining (Fig. 5C). Moreover, the CD133^+^-specific transplanted tumours expressed cytokeratin, which also confirmed their identity as SCC (Fig. 5D).

## Discussion

In this study, we showed that ~0.4% of primary OSCC cells were CD133^+^ cells, which was similar to the 1% to 2% reported previously^24^, and that these CD133^+^ cells showed characteristics of CSCs. Compared with CD133^−^ cells, a large proportion of CD133^+^ cells was in the G0/G1 phase, suggesting that CD133^+^ cells and their daughter cells can re-enter the niche and de-differentiate to replace lost stem cells. Furthermore, CD133^+^ cells not only showed the ability to differentiate into adipose and bone tissues but also a strong tumour-forming ability^14^. The results indicated that the CD133^+^ cell populations displayed greater chemotherapy resistance to cisplatin than autologous CD133^−^ cells, and sphere-formation assays revealed that CD133^+^ cells showed a higher self-renewal ability *in vitro* than CD133^−^ cells. Additionally, we found that the CD133^+^ cell populations showed higher mRNA levels of stemness-associated genes, such as *NANOG*, *SOX2*, *ALDH1A1*, and *OCT4*^25^. Several studies reported that CD133 combined with *OCT4* and *NANOG* expression indicate a poor survival prognosis in OSCC patients, suggesting that these CSC markers are predictive indicators of tumour invasiveness^26^. Therefore, these results demonstrated that CD133^+^ OSCC cells exhibit stem cell characteristics.

MACS is frequently used in cancer and CSC research based on its status as a highly specific cell-separation technique^27,28^. Although several studies report successful CSC isolation using fluorescent-activated cell sorting and other separation techniques, MACS represents a simple and effective approach^29,30^. Previous studies show that CSCs in head and neck SCC express high levels of CD133 and CD44^31^. In the present study, we isolated CD133^+^ cells from primary OSCC cell populations and compared the properties of CD133^+^ and CD133^−^ cells, whereas other studies used CD44/CD133 double-labelled antibody sorting rather than single-labelled antibody sorting of CSCs^31^. However, the present study revealed high levels of CD44 expression (mean: 33.76%) in primary OSCC cells (see Supplementary Table S1 online), which suggested that this CSC marker is less suitable, as many primary OSCC cells express CD44. In the future, stemness maintenance of sorted cells *in vitro* should be further explored.

CSCs can trigger tumour metastasis and resistance to chemotherapy^32^. The first CSCs were discovered in leukaemia, and more recently, studies reported and confirmed the presence of CSCs in other types of cancer, including prostate, liver, breast, and colon, as well as other malignant tumours^33^. The common surface markers currently used to identify CSCs are CD133, CD44, and ALDH1A1, and CSCs are most commonly isolated from OSCC based on their cell-surface markers. Therefore, accurate CSC identification requires a unique surface marker capable of clearly identifying tumour cells. To the best of our knowledge, this is the first report demonstrating that not only OSCC cell lines but also cultured primary OSCC cells expressed CD133, which, as a CSC marker, is associated with the progression of many solid tumours, including those related to melanoma, astrocytoma, and liver cancer^34,35^. Moreover, cancer cells highly expressing CD133 are invasive *in vitro* and associated with poor prognosis in patients^36^. Interestingly, the CD133 expression as a stem cell marker gradually decreases with cell differentiation^37^, which is likely linked to changes in cancer metabolism. CSCs have also been isolated from primary laryngeal cancer cell lines using CD133 as a screening marker^38^. In the present study, we characterised for the first time CSCs in populations of primary OSCC cells using CD133. Other studies suggest that *SOX2* is critically involved, at least in part, in cancer initiation and progression via modulation of CSC stemness^39^. Moreover, a review indicated that *NANOG, ALDH1A1*, and *OCT4* could serve as CSC markers and were predictive of poor prognosis^40^. Therefore, these findings suggest that CD133 can promote the expression of stemness-associated genes. Indeed, our data demonstrated the successful isolation of CD133^+^ cells from primary OSCC cell populations, and that the stemness of CD133^+^ CSCs was stronger than that of CD133^−^ cells. Furthermore, we observed stronger self-renewal potential, colony formation efficiency, adhesive ability, migration and invasive capacities, and mRNA expression of stem cell markers (*NANOG*, *SOX2*, *ALDH1A1*, and *OCT4*) in CD133^+^ cells than in CD133^−^ cells, suggesting that CD133^+^ cells might contribute more to OSCC malignancy.

However, there are limitations to our study. First, a different approach to the sorting of tumour stem cells, such as double-labelled antibody sorting rather than single-labelled antibody sorting, might better reflect CSC characteristics. Second, CD133^+^ and CD133^−^ cells should be tested for their resistance to radiotherapy, and CD133^+^ cells should be tested for their multidirectional differentiation ability, such as chondrogenesis and adipogenesis. Finally, because the *in vivo* microenvironment, including the presence of growth factors, plays an important role in tumour cell growth and tumour formation, it is necessary to study ways to simulate internal environmental factors of human OSCC in tumour formation experiments.

In conclusion, we successfully isolated CD133^+^ cells from primary OSCC cell populations using MACS, with these CD133^+^ cell subpopulations subsequently characterised as CSCs. Our findings provide new insights into the study of OSCC CSCs and valuable cell resources for the development of new treatment strategies for OSCC.

## Methods

### Study design

This study was approved by the Medical Ethics Committee of Guangxi Medical University, and patients and their families were fully informed and provided written consent. The inclusion criteria were as follows: patients with histologically diagnosed OSCC at TNM stage III or IV and with lymphatic metastasis, regardless of the degree of differentiation and whether they received chemotherapy and radiotherapy. The exclusion criteria were the absence of any other communicable disease in patients. The study was performed in accordance with the principles of the revised Declaration of Helsinki (World Medical Association General Assembly, 2008).

### Cell isolation and culture

Specimens were obtained from six patients who underwent OSCC resection at the Affiliated Stomatological Hospital of Guangxi Medical University (Nanning, Guangxi Province, China). Tumour tissues were placed in penicillin/streptomycin solution, transported to the laboratory, rinsed with PBS, cut as thinly as possible with ophthalmic scissors, and eventually cultured in Dulbecco’s modified Eagle medium (DMEM; 319-085-CL; Wisent Biotechnology, Saint-Jean-Baptiste, QC, Canada) containing 14% foetal bovine serum (FBS; Gibco, Gaithersburg, MD, USA) in a 37 °C, 5% CO_2_ incubator. The medium was changed every 2 to 3 days, and cell growth was closely monitored using a light microscope (Olympus Corporation, 67–4 Takakura-machi, Hachioji, Tokyo, 192–0033, Japan).

### MACS separation

According to manufacturer instructions and a previous study^41^, 25 μL of FcR blocking reagent and 25 μL of CD133 microbeads (Miltenyi Biotec, Bergisch Gladbach,130–100–857, Germany) were added to 50 μL of a cell suspension (10^7^ total cells). The OSCC cells were mixed well and incubated at 2 °C to 8 °C for 20 min, followed by washing with 1 mL to 2 mL of the buffer (Miltenyi Biotec) and centrifugation at 1,000 rpm for 5 min. The supernatant was aspirated, and cells were resuspended in up to 500 µL of the buffer, followed by loading of the OSCC cells onto a MACS column (Miltenyi Biotec), which was placed in the magnetic field of a MACS separator (Miltenyi Biotec). The CD133^+^ cells retained in the column and CD133^−^ cells that passed through the column were collected.

### Immunofluorescence staining

Immunofluorescence staining was performed as described previously^42^. Briefly, CD133^+^ and OSCC cells were cultured in 6-well plates with slides for 24 h at 37 °C under 5% CO_2_. The slides were removed and washed with PBS three times, followed by fixation with 4% paraformaldehyde for 20 min, permeabilisation with Tris-buffered saline containing 0.05% Triton X-100 twice, and blocking with 5% FBS for 20 min. The slides were then incubated with a 1:200 dilution of a rabbit polyclonal anti-human CD133 antibody (bioWORLD, Dublin, OH, USA) at 4 °C for 12 h, followed by incubation with a 1:500 dilution of a goat anti-rabbit IgG antibody (bioWORLD) at 37 °C for 1 h and with 4′, 6-diamidino-2-phenylindole (5 µg/mL) at 37 °C for 15 min. Images were captured using a fluorescence microscope (Olympus).

### Flow cytometry analysis

OSCC cells were lysed with trypsin, washed three times with PBS, and then incubated with fluorescein isothiocyanate-conjugated primary mouse anti-human CD133 (BD Biosciences, San Jose, CA, USA) or anti-human CD44 (BD Biosciences) mouse IgG2a (isotype control) antibodies. CD133^+^ cells or CD44^+^ cells were resuspended in 300 µL of PBS, stained with 5 µL of propidium iodide (5 mg/mL) for 30 min, and then analysed by flow cytometry using Cell Quest analysis software (BD Biosciences).

### Cell-differentiation assay

Adipose and osteogenic differentiation of CD133^+^ OSCC cells was induced according to manufacturer instructions (Cyagen, Santa Clara, CA, USA). CD133^+^ cells were cultured in a 6-well plate at a density of 2 × 10^4^ cells in 2 mL of complete medium (Cyagen) per well. The medium was aspirated when cell confluence reached 60% to 70%, and 2 mL of adult bone marrow mesenchyme (Cyagen) was added to each well. Stem cell osteogenic differentiation medium (Cyagen) was changed every 3 days. After induction for 2 to 4 weeks, calcified nodules were fixed with formaldehyde for 40 min and then stained with Alizarin Red. Adipose differentiation was performed in a similar way, with adipose tissue formed by CD133^+^ OSCC cells stained with Oil Red O (Cyagen).

### Cell proliferation assay

The cell proliferation assay was performed as described previously^43^. CD133^+^ and CD133^−^ cells were inoculated into a 96-well plate at 500 cells/well in triplicate and cultured in a 37 °C, 5% CO_2_ incubator. Cell proliferation was measured daily for 7 days. Cell Counting Kit-8 (CCK-8; Dojindo, Tokyo, Japan) solution (10 µL; Vazyme, Nanjing, China) was added to each well, and the plate was incubated in a CO_2_ incubator for 2 h. Absorbance was measured at 450 nm using a microplate reader (BioTek, Winooski, VT, USA). The doubling time (Td) of CD133^+^ and CD133^−^ cells was calculated using the Patterson formula: Td (h) = T × lg2/(lgN2 − lgN1), where N1 represents the incipient absorbance value, N2 is the absorbance value after 7 days of cultivation, and T is the cell proliferation time (h).

### Chemotherapy resistance assay

The chemotherapy resistance assay was performed as described previously^44^. Briefly, CD133^+^ and CD133^−^ cells were trypsinised, and 10,000 cells were placed in 96-well plates with 90 µL DMEM containing FBS. Cisplatin (10 µL; Nippon Kayaku, Tokyo, Japan) was added at different concentrations to the wells in the drug groups, and 10 µL of DMEM containing FBS was added to the negative control group. CCK-8 solution was added to the cells at 100 µL/mL medium 24 h before measurement of cell growth by absorbance at 450 nm using a microplate reader (BioTek). The rate of inhibition of cell proliferation was calculated using the following formula: Inhibition rate (%) = [1 − (A1 − A4)/ (A2 − A3)] × 100, where A1 represents the absorbance value in the drug experimental group, A2 in the blank control group, A3 in the medium only group, and A4 represents the absorbance value of the drug at the same concentration as that used in the experimental group (A1) but without cells.

### Sphere-formation assay

The sphere-formation assay was performed as described previously^33^. CD133^+^ and CD133^−^ cells (1,000 cells/mL) were cultured in 6-well plates (Corning, Corning, NY, USA) in serum-free DMEM-F12 (Wisent Biotechnology) supplemented with B27 (1:50; Invitrogen, Carlsbad, CA, USA), 20 ng/mL epidermal growth factor (BD Biosciences), 0.4% bovine serum albumin (BSA; Wisent Biotechnology), and 5 mg/mL insulin (Sigma–Aldrich, St. Louis, MO, USA). After 2 weeks, spheroid size and number were analysed under a light microscope and those with a diameter >75 µm were counted.

### Semi-quantitative RT-PCR

RT-PCR analysis was performed as described previously^45^. Total RNA was extracted from CD133^+^ and CD133^−^ OSCC cells using RNAiso Plus reagent (Takara, Shiga, Japan), with RNA purity and concentration determined using a NanoDrop 1000 spectrophotometer (Thermo Fisher Scientific, Wilmington, DE, USA). cDNA was synthesised from 1 µg of RNA using the PrimeScript RT reagent kit with gDNA Eraser (Takara). The following forward and reverse primers were used, respectively: 5′-CTTGAATCCCGAATGGAAAGGG-3′ and 5′-GTGTATATCCCAGGGTGATCCTC-3′ for *OCT4*; 5′-GCCCTGCAGTACAACTCCAT-3′ and 5′-GACTTGACCACCGAACCCAT-3′ for *SOX2*; 5′-GTCCCAAAGGCAAACAACCC-3′ and 5′-GCTGGGTGGAAGAGAACACA-3′ for *NANOG*; 5′-TTGGAATTTCCCGTTGGTTA-3′ and 5′-CTGTAGGCCCATAACCAGGA-3′ for *ALDH1A1*; and 5′-GAAGGTGAAGGTCGGAGTC-3′ and 5′-GAAGATGGTGATGGGATTTC-3′ for *GAPDH*. The reaction was performed in a 7500 real-time PCR system (Life Technologies, Carlsbad, CA, USA) using SYBR Premix Ex Taq (Takara) and the following thermocycling conditions for all genes: denaturation at 95 °C for 30 s, followed by 40 cycles at 95 °C for 5 s and 60 °C for 34 s. PCR products were separated on a 1.0% agarose gel and visualised using LightCycler 480 SYBR Green I (Roche, Basel, Switzerland). Band intensities were determined, and their ratios to that of *GAPDH* were calculated using ImageJ software (v.2.1.4.7; National Institutes of Health, Bethesda, MD, USA).

### ***In vivo*tumourigenic assay**

BALB/c mice (4 weeks old) were purchased from the Guangxi Medical University Laboratory Animal Centre, and experiments were conducted in accordance with the guidelines of the ethics committee. After reaching confluence in DMEM, CD133^+^ cells (1 × 10^4^) and CD133^−^ cells (1 × 10^5^) were resuspended in 200 μL of the medium and subcutaneously injected into the BALB/c mice. The mice were sacrificed after 21 days, and tumour histology was analysed.

### Immunohistochemical staining

Immunohistochemical staining was performed according to a previously described protocol, with minor modifications^46^. Tumour model samples were processed as formalin-fixed

paraffin-embedded tissue blocks, The sections were then cut into 4-µm-thick sections and dewaxed in xylene, rehydrated using an ethanol gradient in water, and rinsed with PBS. Antigen retrieval was performed by heating the sections with a sodium citrate solution in a 95 °C water bath for 15 min. The cut sections were treated with 3% hydrogen peroxide and blocked with BSA for 30 min. The sections were incubated overnight at 4 °C with monoclonal rabbit anti-cytokeratin (1:250; cat. no. BH0149; Bioss, Beijing, China). Thereafter, the slides were incubated with an undiluted polymer helper (cat. no. PV-9000; ZSGB-BIO, OriGene Technologies, Beijing, China) for 20 min at 37 °C, followed by staining with appropriate undiluted secondary antibodies (rabbit, polyperoxidase-conjugated; ZSGB-BIO, OriGene Technologies) for 20 min at 37 °C. Colour was developed using diaminobenzidine as a chromogen. Slides were assessed and scored by pathologists under a light microscope (Olympus).

### Statistical analysis

All analyses were performed using SPSS 22.0 software (IBM Corp., Armonk, NY, USA). Data are expressed as the mean ± standard deviation. A Student’s *t* test was used to compare differences prior to and following treatment. Data were considered statistically significant at *P* < 0.05.

## Supplementary information

Supplementary information.
