# Supplementary material for: Characterisation of a subpopulation of CD133+ cancer stem cells from Chinese patients with oral squamous cell carcinoma
Source: Sci Rep. 2020 Jun 1;10:8875. doi: 10.1038/s41598-020-64947-9 (PMC7264286; doi:10.1038/s41598-020-64947-9)
Supplement: Supplementary file 1 — Supplementary information. [file 41598_2020_64947_MOESM1_ESM.docx]

**Supplementary Information**

**Characterisation of a subpopulation of CD133^+^ cancer stem cells from Chinese patients with oral squamous cell carcinoma**

Zhen Ma^1,2,3,4,+^, Chong Zhang^1.2.3.4+^, Xiaotong Liu^1^, Fang Fang^1^, Shiqi Liu^1^, Xianxiang Liao^1^, Shicheng Tao^1^, Huaming Mai^1,2,3,4,*^

**Supplementary Table S1.** Information concerning the six Chinese patients participating in this study.

| Case | Age (y) | Gender | Lesion site | Histological grade | Clinical stage | CD133 (%) | CD44 (%) |
| --- | --- | --- | --- | --- | --- | --- | --- |
| 1 | 44 | Male | Tongue | Well- differentiated OSCC | III | 0.49 | 11.85 |
| 2 | 29 | Male | Tongue | Moderately differentiated  OSCC | III | 0.42 | 39.49 |
| 3 | 38 | Male | Palate | Well-differentiated  OSCC | IV | 0.32 | 8.15 |
| 4 | 48 | Female | Gingival | Well-differentiated  OSCC | IV | 0.43 | 28.00 |
| 5 | 42 | Male | Tongue | Moderately differentiated OSCC | IV | 0.35 | 36.60 |
| 6 | 49 | Male | Tongue | Moderately differentiated  OSCC | III | 0.45 | 78.50 |
